# Talking sensibly about depression

**DOI:** 10.1371/journal.pmed.1002257

**Published:** 2017-04-04

**Authors:** Vikram Patel

**Affiliations:** 1 London School of Hygiene & Tropical Medicine, London, United Kingdom; 2 Public Health Foundation of India, National Capital Region of New Delhi, India; 3 Sangath, Goa, Porvorim, India

## Abstract

In an Essay to highlight World Health Day 2017, Vikram Patel proposes a staged model, from wellness to distress to disorder, for classifying depressive symptoms.

**Summary points**

- Depression is the leading mental health—related cause of the global burden of disease and is the focus of World Health Day 2017.
- Despite robust evidence of the effectiveness of interventions ranging from self-care to clinical interventions, the vast majority of people with depressive symptoms do not receive any care.
- The majority of persons meeting the binary diagnostic criteria for depression have mild to moderate symptoms that most often do not need clinical interventions.
- A staged model, from wellness to distress to disorder, offers a hybrid between binary and dimensional approaches to classifying depressive symptoms, with specific interventions delivered through distinct delivery platforms addressing each stage.
- Such a staged approach is likely to be more efficient and acceptable to diverse audiences (from the general population to policy makers and practitioners) and provides the basis for these audiences to talk with one voice about this condition.

The past 12 months have been a momentous year for global mental health, the discipline of global health concerned with reducing the burden of mental ill-health and inequalities within and between nations. The World Bank and WHO jointly hosted the “Out of the Shadows” summit in Washington in April 2016 to highlight mental health as a global “development” priority, signifying a notable shift in emphasis from a narrower health focus to development more broadly. Twelve months later, the WHO celebrates its annual World Health Day in April 2017 on the theme of depression. This is a very timely (indeed, greatly overdue) recognition, for not only is depression the leading mental health—related cause of the global burden of disease but also because, despite the reams of evidence on how the suffering associated with this condition can be mitigated, vast proportions of people globally do not benefit from these interventions [1]. Even in the relatively better-resourced, middle-income countries such as India and China, up to 90% of patients with depression report not having sought or received any care for their symptoms [2].

The slogan for World Health Day is “let’s talk,” emphasizing the central role of disclosure “as a vital component of recovery” by targeting the stigma surrounding mental illness, which acts as a barrier to people with depression seeking help. Significantly, the WHO campaign recommends that talking can involve a wide range of potential listeners, from family members and friends to health professionals, as well as encouraging open discussions about this condition in settings such as schools, the workplace, and in the media, “ultimately leading to more people seeking help.” I emphatically support the notion that seeking help must include both professional and nonprofessional actors. Despite this pragmatic recommendation, there is still little tangible action by governments and health systems to implement the evidence on effective interventions, and this is, at least in part, because of dissonant perspectives about the very nature of this condition. Indeed, some commentators view the discourse on the global burden of depression and the treatment gap as a culturally insensitive plot to export a failed psychiatric model to unsuspecting developing countries and a ploy to expand markets for pharmaceutical companies [3].

If we are to talk sensibly about depression, one must explicitly acknowledge that the term itself captures a very heterogeneous group of experiences, at least some of which can be addressed by “talking” with friends, while at other times, one may need a health professional. A major challenge to acknowledging this fundamental diversity of the experiences of depression is the current approach to the classification of the condition, which is, inadvertently, contributing to the large “treatment gaps” and the clash of ideas [4].

## The classification of depression

The past two decades have witnessed the production of a large evidence base from diverse disciplinary perspectives on the human experience that psychiatry classifies as depression. The fundamental problem with this diagnosis is that, in mimicking the model used for classifying other health conditions, a binary classification has been imposed on the continuum of mood to distinguish “cases” from “noncases.” This binary model is unsuitable for depression because there is no clear defining line which discriminates between the miseries of daily life from the “disorder” that can benefit from a clinical intervention. While the hunt for a biomarker to enable the accurate discrimination of those individuals who may require clinical interventions continues apace, there are no promising leads on the horizon. Further, given there is no obvious point in the distribution of symptoms of depression that demarcates the “well” from the “ill,” it is highly unlikely that we will ever find such a biomarker that can neatly distinguish those who are “depressed” from the rest of the population. Thus, the current approach, constrained by binary models of defining when a person may have a disorder, must be content with relying entirely on eliciting symptoms related to the inner emotional worlds of a person (the hallmark ones being feeling miserable, losing interest in things, and feeling profoundly fatigued), assessing the duration and impact of these symptoms, and, based on an arbitrary algorithm, using this information to arrive at a “diagnosis.” Critiques of the binary approach have pointed out that it risks medicalizing normative human responses to adversity and loss (a “category fallacy”) and that applying a binary categorization to a phenomenon that is so obviously continuously distributed in the population is fundamentally unsound [5]. A dimensional approach, consistent with that proposed by the National Institute for Mental Health’s Research Domain Operating Criteria [6], is proposed as a more valid alternative. However, while dimensions are useful for social and neuroscientists, categories have the greatest utility for health workers and policy makers. Thinking dimensionally helps one understand problems, whereas acting categorically helps one solve them. Both matter to people who are experiencing depressive symptoms. One potential way forwards to find a balance between these two poles is by modifying the binary model into an ordinal one, a hybrid equivalent of the Likert scale, from wellness through distress and disorder of increasing severity or chronicity (Table 1).

**Table 1:** The stages of the depressive symptom continuum.

Stage	Definition	Focus of intervention	Platform of care	Interventions
Wellness	Absence of any sustained, distressing, emotional experiences	Promotion and primary prevention	Community	Promoting nurturing environments for children and adolescents, life skills on promoting mental health, addressing social determinants such as interpersonal violence, etc.
Distress	Mild to moderate distressing emotional experiences, of relatively short duration	Indicated prevention	Community, routine health care*, social welfare	Self-care and low-intensity support through digital or peer interventions
Depressive disorder	Severely distressing experiences, lasting at least two to four weeks, with impairment of social functioning	Treatment to remission and recovery	Routine health care	Brief psychological treatments, antidepressant medication, or a combination of both, in a stepped care approach
Recurrent or refractory depressive disorder	Unresponsive or relapsing depressive episodes	Relapse prevention and/or stabilization	Mental health care	Intensive psychosocial interventions, augmented pharmacotherapy, electroconvulsive therapy

*Routine health care refers to any health care platform, other than mental health care, where depression is frequent and includes primary, maternal, and chronic disease care.

## A staged model of depression

A hybrid model is consistent with the “staging” of psychiatric disorders, advocated for more than a decade [7], in response to the concerns that binary diagnoses mask the heterogeneity of presentations and trajectories within each diagnostic category, and the consequent “one size fits all” approach leads to under- or overtreatment of a significant proportion of individuals with the diagnosis. Indeed, one of the key merits of staging is its direct relevance to the selection of appropriate interventions (Table 1). In the recent trial of the Healthy Activity Program (HAP), a brief psychological treatment based on behavioural activation for severe depression in primary care in India, up to 90% of individuals with depressive symptoms meeting binary criteria for a diagnosis had mild to moderate severity [8]. The staging approach would recategorize these individuals as having a distress syndrome (typically, a mixture of mood, anxiety, and somatic symptoms). The majority of these individuals would do just as well with low-intensity interventions such as self-care, web-based psychological therapy, and social support as with clinical interventions [9]; indeed, baseline severity is the most consistent moderator of the effectiveness of clinical interventions, which are mostly effective for the severe forms of depression [10,11].

The role of the health care system would be to triage people who are distressed to receive low-intensity interventions in the community. This may be considered “preemptive” care, contributing not only to improved population mental health and reduced incidence of disorders but also enabling the clinical sector to attend to those with severe disorders. This approach also serves to enhance the central role of the affected person in the recovery process, empowered with adequate knowledge and support. It is important to emphasize that people who are distressed are not to be dismissed as the “worried well,” for this group contributes, from a public health perspective, to a significant fraction of the burden of impairment associated with depressive symptoms, and a proportion of these individuals may progress to develop a frank depressive episode or fail to respond to low-intensity interventions [12]; these will need to “graduate” to the next stage requiring clinical interventions. The point is to emphasize is that the majority of distressed individuals do not need a biomedical label and their intervention need not be medicalized.

At the next stage of this distribution, those who have severe symptoms that would be considered to meet the criteria for disorders (about 10% of the total burden of depressive symptoms in primary care in the HAP trial), there is no ambiguity in the evidence that clinical interventions are cost-effective, and the emphasis must be to scale up these interventions. While many health care systems and medical practitioners, particularly in low- and middle-income countries, have interpreted clinical interventions as being equivalent to antidepressant medication, in fact the strongest evidence from the global context is for brief psychological treatments. These treatments, largely based on cognitive—behavioural and interpersonal theoretical frameworks, are typically delivered by nonprofessional frontline workers (such as community health workers) in primary care or community-based settings in six to ten sessions over two to three months. A large body of evidence—including 27 trials in a recent review [13] and bolstered by several more recent trials [8,14]—testifies to the high levels of acceptability, large effects, and good value for money that these interventions offer. The scaling up of these treatments through routine primary care or other health care platforms (notably maternal health care, for which an equally impressive body of evidence demonstrates the effectiveness of nonspecialist-delivered psychological treatments [15]) should now focus on integrating the management of depressive disorder with other common mental disorders (anxiety disorders and somatoform disorders) through transdiagnostic interventions [16] and, more broadly, with other chronic conditions.

The final, most severe stage is when the depressive disorder does not respond to clinical interventions in primary care or runs a recurrent or relapsing course (approximately a third of individuals with depression in the HAP trial or in the large United States Sequenced Treatment Alternatives to Relieve Depression [STAR*D] trial, which evaluated a sequence of clinical interventions [17]). This group of patients who may be referred to as “chronic” or “refractory” and comprise a relatively small number of patients (under 5% of patients in primary care with any depressive symptoms in the HAP trial) are the most impaired functionally and should be the focus of specialist clinical interventions such as intensive psychosocial treatments, augmented pharmacotherapy, or ECT (the latter being especially indicated for patients with psychotic features, with severe self-neglect or very high risk of self-harm) [1].

## A pragmatic care system for depression

A key barrier to implementing this staged model and integration of the management of a depressive episode in routine health care is the very low rates of detection of these disorders. The traditional approach to improve detection has been through training of primary care workers, despite decades of frontline experience showing that this approach has little sustainable effect. Recent randomized controlled trials in low- and middle-income countries further confirm the limited value of training alone [18,19]. While the low detection rates are likely to be the result of multiple factors, including the lack of skills or resources to respond to a diagnosis and the fear of being overwhelmed by more work, we must recognize that training alone is an inadequate intervention for improving detection. An alternative may be to incorporate screening of adults in primary care and maternal health care platforms using locally validated symptom measures such as the Patient Health Questionnaire-9 (PHQ-9), as has been recommended by the US Task Force on prevention [20]. These measures are brief, acceptable to patients, and their scalability can be enhanced by delivery in graded steps (e.g., asking two “core” questions of the PHQ-9 to all patients and the remainder only to those who respond positively to at least one) or digitally (e.g., through apps while the patient is waiting to see the health worker). Moreover, these tools can also be used to track clinical progress (including remotely through smartphone apps) and for remote supervision of frontline workers, as has been done with high levels of acceptability in the United Kingdom’s Improving Access to Psychological Treatments program [21]. However, screening must always be seen as only the first step of a comprehensive depression care program, for screening alone without any strategies to ensure appropriate response to the results may have limited impact on patient outcomes [22]. Further, the cost-effectiveness of screening may be undermined by false positives [23], although this limitation may be mitigated by the staging approach.

Following detection, and after triaging out distressed persons to low-intensity interventions, those with a depressive episode should be offered either brief psychological treatments or antidepressant medication, both of which should be made available in routine care. The former could be delivered by the same health worker who acts as a care manager, the critical human resource needed to coordinate the collaborative care, which is the most proven delivery model for integrating depression management in routine care [24]. Notably, amongst the effective psychological treatments for depression is behavioural activation, an approach which has conceptual alignment with activity-promotion interventions typically advocated for other common chronic conditions such as diabetes, indicating the potential synergies inherent in integrating care for these diverse conditions, in addition to the well-established rationale of the high prevalence of multiple morbidities and the bidirectional causal pathways between these morbidities [25]. Another key shared feature of care of diverse chronic conditions is the need for a stepped care paradigm with proactive tracking of patient outcomes to identify nonresponders or relapses early and to “step up” (e.g., in the case of depression, through combined drug—psychotherapy protocols or referral of relapsing and/or refractory episodes for specialist interventions [25]).

## Reducing the global burden of depression

Reorienting the binary diagnostic model currently in use towards a more nuanced hybrid categorical-dimensional staged model can address several barriers facing global mental health. The first is reducing the potential numbers of people who need clinical interventions. Given that the population point prevalence of depression, based on the binary system, is estimated at about 5% of the adult population [26]—translating to over 200 million people globally—few countries, particularly in the global south, have a health care system that can cope with the massive numbers of people who would meet the current diagnostic criteria. The staged model would dramatically reduce the estimates of the numbers of persons with depressive symptoms who need clinical interventions, offering a better prospect of reducing the treatment gap for disorders both by reducing the denominator of this fraction and by focusing the energies of the health care system to detect and treat these disorders.

Second, a staged model can contribute to greater attention to prevention across the continuum of promotion, prevention, treatment, and rehabilitation. The opportunities and evidence base for prevention have mostly been focused on indicated prevention—i.e., interventions targeted at individuals who are distressed (also referred to in the binary classificatory system as persons with “subthreshold” symptoms), which are typically low-intensity versions of cognitive—behavioural interventions [27]. This is not surprising, as it is rarely feasible to mount the kind of long-term studies needed to demonstrate the impact of interventions addressing distal social determinants, such as those aiming to promote nurturing environments (e.g., through universal early child stimulation and selective prevention for children facing social deprivation) on reducing the incidence of depression. However, it is highly likely, given the strong observational evidence linking social determinants such as childhood adversity and low educational attainment with the risk of depression in adulthood [28], that interventions targeting early life course influences are likely to have significant impacts on the primary prevention of depression. In the more distal life course phases of youth, building emotional and social competencies and enabling nurturing school and community environments also have a growing evidence base to support their role in prevention [1]. The goal of these interventions is to ensure that most of the population remains in the well-being stage.

Thirdly, such a shift may enable us to “talk” more openly about depression, as it addresses fear in the community that such talk automatically implies a disorder demanding clinical interventions from a mental health specialist. Indeed, it can help reduce the yawning credibility gap—i.e., the gap between the improbably large numbers thrown up by epidemiological surveys that define “cases” based on current binary classifications and the views and concepts held by most other audiences, including the community at large and primary care practitioners (who, often with good reason, are reluctant to deploy clinical interventions to such large numbers of patients whose depressive symptoms may be interpreted as being normative or transient) [29]. A key lesson from programs seeking to enhance access to care and the acceptability of psychological treatments for depression in the global context is that most innovators avoid psychiatric labels in favour of contextually informed metaphors and explanations [30]. Idioms such as “thinking too much” in many African cultures and “tension” in India are less stigmatizing and widely understood, and they seamlessly capture the continuum of distress and disorder. Such approaches, which are aligned with the idiom of distress, can lead to a dramatic increase in demand for care, an essential prerequisite to ensuring that people move from more severe stages to milder ones and ultimately towards wellbeing [31].

## “Let’s talk” sensibly

It is becoming increasingly commonplace, even trendy perhaps, to talk about depression, not least due to the growing number of celebrities, from Bruce Springsteen to Deepika Padukone, disclosing their personal experiences of struggle and recovery. However, to move this discourse beyond celebrities to the general population, in particular amongst those experiencing social adversities who are disproportionately affected by depressive symptoms, we need to move from a binary classification to a staged model that explicitly recognizes the dimensional nature of this condition. Such a revised framing has potential utility for diverse audiences, including scientists, policy makers, patients, and practitioners, and offers a framework for consensus between diverse disciplines, between the clinical and public health communities, and between professionals and civil society on how to talk sensibly about depression, in one voice. There is no doubt whatsoever that we must talk about depression more openly, but we must ensure that people experiencing depressive symptoms are always at the heart of the discourse.

## Abbreviations

HAP: Healthy Activity Program
PHQ-9: Patient Health Questionnaire-9
